# Effect of Sodium Metabisulfite on Physicochemical Indexes and 16S rRNA-Based Microbial Communities of Fresh-Cut Potatoes During Chilled Storage

**DOI:** 10.3390/foods15142426

**Published:** 2026-07-08

**Authors:** Zhengnan Ren, Lin Zhou, Lele Zhao, Tingting Yang, Binbin Li, Xiaoying Guo, Xinhui Wang, Longquan Xiao

**Affiliations:** 1 School of Food and Biological Engineering, Chengdu University, Chengdu 610106, China; rzhengnan@outlook.con (Z.R.);; 2 Sichuan Kelun Pharmaceutical Co., Ltd., Chengdu 610599, China; 3 Local Financial Funds of National Agricultural Science and Technology Center, Chengdu 610213, China

**Keywords:** fresh-cut potatoes, sodium metabisulfite, physicochemical indexes, bacterial diversity

## Abstract

Browning and microbial spoilage are major constraints on the shelf-life of fresh-cut potatoes, yet the concentration-dependent effects of sodium metabisulfite (SM) on product quality and bacterial dynamics are poorly understood. In this study, the impact of SM on the quality and bacterial dynamics of fresh-cut potatoes during storage was investigated. This study evaluated the concentration-dependent effects of SM on the quality of fresh-cut potatoes. The results showed that 0.3% SM treatment effectively delayed browning. Subsequently, the quality and bacterial community of fresh-cut potatoes of CK and 0.3% SM-treated groups were further investigated. The results indicated that a 0.3% SM treatment could inhibit the activities of polyphenol oxidase (PPO), peroxidase (POD) and phenylalanine ammonia-lyase (PAL) as well as the growth of *Duganella*. On day 7 of storage, the L* values of the control group (CK) and 0.3% SM groups decreased by 17.02% and 7.68%, respectively, while their total plate counts were 4.33 and 4.21 log CFU/g, showing a significant difference between the two groups (*p* < 0.05). Furthermore, SM treatment extended the shelf-life of fresh-cut potatoes to approximately 7 days, decreased the loss of soluble solids and weight, and maintained textural characteristics (firmness and elasticity). These findings demonstrate that SM treatment is an effective strategy for preserving the quality of fresh-cut potatoes and provide new insights into its role in inhibiting enzymatic browning during storage.

## 1. Introduction

Potato (*Solanum tuberosum* L.) ranks among the four primary staple crops worldwide, accounting for approximately 26% of the world’s total output [1]. Potato contains high levels of carbohydrates and proteins, and is also valued for its abundant dietary fiber, minerals, and vitamins, which can prevent cardiovascular and cancer-related diseases [2]. With the ever-increasing speed of modern life, fresh-cut potato occupies a large proportion of people’s diets due to convenient processing and rich nutrition. However, fresh-cut potatoes are susceptible to browning, which significantly impairs their sensory quality. Therefore, it is crucial to explore strategies and mechanisms for suppressing discoloration in fresh-cut potatoes.

In recent years, various methods, such as ultrasound, high pressure, microwave treatment, edible coatings, and anti-browning agents, have been employed to inhibit the browning reaction; however, these methods often suffer from high costs and low efficiency [3]. Sodium metabisulfite (SM) is commonly employed to prevent browning in freshly cut produce owing to its low cost and operational simplicity. It suppresses non-enzymatic browning, various enzymatic reactions, and microbial growth, while also serving as a pH regulator and stabilizer [4]. Due to its antimicrobial activity, color stabilization, anti-browning, and antioxidant effects, SM is widely applied as a food additive; SM is Generally Recognized as Safe (GRAS) by the FDA for food preservation [5]. It can be incorporated into dried fruits and vegetables, seafood, fruit juices, as well as alcoholic and non-alcoholic drinks [6]. It also exhibits a strong preservative effect on Chinese herbs [7]. Previous studies on SM application in fresh-cut produce have primarily focused on sensory quality and enzymatic browning inhibition. For instance, Nascimento demonstrated that the browning reaction of ready-to-eat processed potatoes treated with SM (2.5%) and ascorbic acid (2.5%) under vacuum packaging was effectively inhibited. Utama et al. reported that SM and L-arginine significantly inhibited browning in fresh-cut apples during storage at 10 °C [8]. However, these studies mainly evaluated macroscopic quality attributes without investigating the underlying microbiological changes. More importantly, the effect of SM on the bacterial community composition and dynamics in fresh-cut produce during cold storage (4 °C) has not been examined to date. While SM is known to possess antimicrobial activity, its impact on the spoilage-related microbial community structure—rather than merely total microbial counts—remains unexplored. This represents a critical knowledge gap because browning and quality deterioration in fresh-cut potatoes are not solely enzymatic processes, they are also closely associated with microbial proliferation and metabolic activities that can accelerate tissue damage and discoloration.

In this study, the impact of varying SM concentrations on the appearance quality, color, and browning degree of samples during chilled storage was investigated. Then, the optimal SM concentration was selected, and on this basis, the effects of SM treatments on sensory evaluation, weight loss, pH, soluble solids, malondialdehyde (MDA) content, texture, PPO, POD, PAL, total plate count, and bacterial community of fresh-cut potatoes during chilled storage were investigated.

## 2. Materials and Methods

### 2.1. Reagents and Chemicals

Trichloroacetic acid (AR), Macklin Biochemical Co., Ltd. (Shanghai, China); Thiobarbituric acid (AR), Sinopharm Chemical Reagent Co., Ltd. (Shanghai, China); Sodium hydroxide (AR), Fuchen (Tianjin, China) Chemical Reagent Co., Ltd. (Tianjin, China); Food-grade vegetable preservation bags (17 × 25 cm), Dongguan Xinrunlong Packaging Material Co., Ltd. (Dongguan, China); Plate count agar medium (BR), Hangzhou Microbial Reagent Co., Ltd. (Hangzhou, China); Sodium chloride (AR), Tianjin Zhonglian Chemical Reagent Co., Ltd. (Tianjin, China); Polyphenol oxidase assay kit, Peroxidase assay kit, Phenylalanine ammonia-lyase assay kit, Nanjing Jiancheng Bioengineering Institute (Nanjing, China).

### 2.2. Sample Processing

Potatoes (*Solanum tuberosum* ‘Chuanyu 16’) were purchased from a local supermarket (Longquanyi, Chengdu, China) and obtained from a single batch. Potatoes free of pests and diseases, without mechanical damage and of uniform size were selected, cleaned, peeled and sliced to a thickness of 4–5 mm. Based on preliminary experiments, the fresh-cut potatoes were soaked in 0.1%, 0.3%, and 0.5% SM solutions for 5 min, then rinsed with clean water to eliminate any residual SM, and stored in polypropylene food preservation bags at 4 °C, and the fresh-cut potatoes soaked with clear water were named CK as control group. A total of 270 slices of potatoes were taken and 60 slices (4 groups × 5 times × 3 technical replicates) were sampled at 0, 1, 3, 5 and 7 days of storage for color and browning indices and screening for suitable SM concentration. Thirty samples (2 groups × 5 times × 3 technical replicates) were taken for sensory scoring and texture of SM fresh potatoes, thirty samples (2 groups × 5 times × 3 technical replicates) were taken for determination of pH, soluble solids, and MDA content, thirty samples (2 groups × 5 times × 3 technical replicates) were used to determine weight loss, thirty samples (2 groups × 5 times × 3 technical replicates) were used to determine colony size, and 30 samples (2 groups × 5 times × 3 technical replicates) were used to determine enzymes. Thirty samples (2 groups × 5 times × 3 technical replicates) were used to determine the enzyme activity and, finally, thirty samples (2 groups × 5 times × 3 technical replicates) were used to determine the bacterial community.

### 2.3. Sensory Evaluation

Sensory evaluation was conducted following the method described by Wang et al. with a slight modification. [9]. The samples were evaluated by nine trained panelists on 0, 1, 3, 5, and 7 days of storage. Fresh-cut potatoes were evaluated on four indicators: color, flavor, texture and spoilage, mainly using a scoring system (5 = very good, 4 = good, 3 = fair, 2 = poor, 1 = very poor), with the average being the final total score.

### 2.4. Weight Loss

Weight loss was determined as follows and calculated as follows:

$$Weight\;(\%)=\frac{W_{0}-W_{1}}{W_{0}}\times 100\%$$

where W_0_ refers to the original weight and W_1_ is the weight on the day of sampling.

### 2.5. Color Value

Potato slices color (L*, a*, and b*) was assessed by a convenient hand-held colorimeter (CR-400 Colorimeter, Konica Minolta, Seoul, Republic of Korea); each sample was randomly obtained from five locations.

### 2.6. pH Value

The pH was analyzed based on the procedure described by Keydis Martínez et al. [10] with a slight modification. In short, a pH meter (Testo205, Shenzhen Testo Instrument Co., Ltd., Shenzhen, China) was used to detect the pH of the supernatant after 10 g of samples were pulped and filtered.

### 2.7. Determination of Browning Degree (BD)

Browning degree was calculated using the methodology established by Sun et al. [11] with minor modifications. Ten milliliters of cold water were used to homogenize two grams of potato tissue samples. The supernatant was kept after centrifuging at 4500 rpm for 15 min at 4 °C. A spectrophotometer (UV-5200, Shanghai Yuan Analytical Instrument Co., Shanghai, China) was used to measure absorbance at A410 nm.

### 2.8. Texture Analysis

Texture analysis was conducted following the method of Zhou et al. [12] with minor modifications. The sample was subjected to textural analysis using a P/6 probe (TA.XTplusC Stable Micro Systems Ltd., Surrey, UK) with the following parameter settings: compression ratio 30%, trigger force 5 g, pre-test speed 1.0 mm/s, mid-test speed 1.0 mm/s, and post-test speed 5.0 mm/s. Eight measurements were made of each sample, and the mean values were determined.

### 2.9. Soluble Solids Content

Soluble solids were conducted using the protocol of Wang et al. [13] with a slight modification. Briefly, 10 g of samples was ground in a mortar and filtered. The filtrate was assayed using a handheld saccharimeter (PAL-1, ATAGO, Tokyo, Japan).

### 2.10. MDA Content

MDA content was measured following the method described by Sajid Ali et al. [14] with a slight modification. In short, 1 g of tissue was homogenized in 5 mL of trichloroacetic acid and centrifuged at 13,000 rpm for 7 min. Then, 2 mL of the supernatant was mixed with an equal volume of thiobarbituric acid; the mixture was then heated at 100 °C for 20 min, cooled to room temperature, and the absorbance was determined at 532, 450, and 600 nm. MDA content was calculated using the following formula:

MDA (μmol/g) = 6.45 × (A_532_ − A_600_) − 0.56 × A_450_

where A_450_, A_532_ and A_600_ were the absorbance values at 450, 532 and 600 nm, respectively.

### 2.11. PPO Activity

PPO activity was assayed using a PPO kit (Nanjing Jiancheng Bioengineering Institute, Nanjing, China). One gram of the sample was mixed with 5 mL of extraction solution and homogenization in an ice bath. The mixture was subsequently centrifuged at 8000 rpm for 10 min under ambient conditions, and the supernatant was collected and measured at a wavelength of 420 nm using a visible spectrophotometer.

### 2.12. POD Activity

A POD kit (Nanjing Jiancheng Bioengineering Institute, Nanjing, China) was used to measure POD activity. A 10% tissue homogenate was produced in an ice bath by adding one gram of the sample to nine milliliters of a 0.9% NaCl aqueous solution. After centrifuging the mixture for ten minutes at 3500 rpm, the supernatant was collected and quantified using a visible spectrophotometer at a wavelength of 420 nm.

### 2.13. PAL Activity

A PAL kit (Nanjing Jiancheng Bioengineering Institute, Nanjing, China) was used to measure PAL activity. After adding one gram of sample to nine milliliters of extraction solution, the mixture was homogenized for ten minutes at 10,000 rpm in a cold-water bath. After centrifuging the mixture for ten minutes, a visible spectrophotometer was used to measure the supernatant at 290 nm.

### 2.14. Determination of the Total Plate Count

Using a homogenizer (PJ200-SH, Shanghai Specimen Model Manufacturing, Shanghai, China), a 10 g potato slice sample was aseptically removed from each package and placed into a homogenizer bag containing 90 mL of sterile saline. The mixture was then homogenized, and 1 mL of the resulting homogenate was diluted tenfold with 9 mL of saline. Next, 1 mL of this dilution was combined with plate counting agar (Shanghai Bo Microbial Technology Co., Ltd., Shanghai, China) and incubated at 37 ± 1 °C for 48 h. The total plate count was recorded and expressed as log CFU/g.

### 2.15. Analysis of Microbial Diversity

DNA extraction was performed from the samples by CTAB/SDS technique and DNA concentration was detected. 16S V3–V4 region (341F: ACTCCTACGGGAGGCAGCAG and 806R: 5′-GGACTACHVGGGTWTCTAAT-3′) was used for amplification, under the following conditions: initial denaturation at 98 °C for 1 min, then 30 cycles of 98 °C for 10 s, 50 °C for 30 s, and 72 °C for 30 s. The last cycle was kept at 72 °C for 5 min. The resulting amplified sequences were analyzed using the Illumina HiSeq platform. Barcode and primer sequences were first removed, and the reads from each sample were subsequently assembled with FLASH (version 1.2.11). The assembled raw tags were then subjected to stringent quality filtering using fastp (version 0.23.1) to generate high-quality clean tags [15], and chimeric sequences were eliminated to obtain the final valid data. Representative sequences of the annotated OTUs were analyzed for species identification using the Mothur method and the SILVA SSU rRNA database, with thresholds set between 0.8 and 1.0, to obtain taxonomic information at various levels, and community composition for each sample based on species count. PyNAST software (v 1.2.2) and GreenGene database “Core Set” data information were used for rapid multiple sequence comparison to obtain the systematic relationships of all OTU representative sequences, the sample data were standardized and unified [16]. To evaluate differences in species complexity among groups, alpha diversity indices were computed using QIIME software (version 1.9.1) [17]. Beta diversity analysis was conducted using QIIME to assess the compositional complexity of microbial communities and to compare inter-group differences, based on unweighted UniFrac distances. In this study, three biological replicates were analyzed per group. The raw 16S rRNA amplicon sequencing data from this study are available at the China National GeneBank Database (CNGB) under accession number CNP0009721.

### 2.16. Residual SO_2_ Content

The residue from the use of sodium metabisulfite is sulfur dioxide (SO_2_). Determination of SO_2_ was performed following the acid-base titration procedure outlined in the Chinese Standard (GB/T 5009.34-2022) [18], and the measured results are expressed in mg/kg.

### 2.17. Statistical Analysis

Each experiment was carried out in triplicate or more, and all data are reported as mean ± standard deviation. Microsoft Excel 2021 was used for data processing. Data obtained at each storage time point were analyzed separately. For comparisons among three or more groups, one-way analysis of variance (ANOVA) was performed, followed by Duncan’s multiple range test for post hoc pairwise comparisons. For comparisons between two groups, Student’s *t*-test was applied. Differences were considered statistically significant when *p* < 0.05. Origin 2024 software (2024b, Origin Lab Corporation, Northampton, MA, USA) was utilized for graphing.

## 3. Results and Discussion

### 3.1. Effects of SM at Different Concentrations on the Color, Browning Degree and Appearance Quality of Fresh-Cut Potatoes

**Figure 1:**
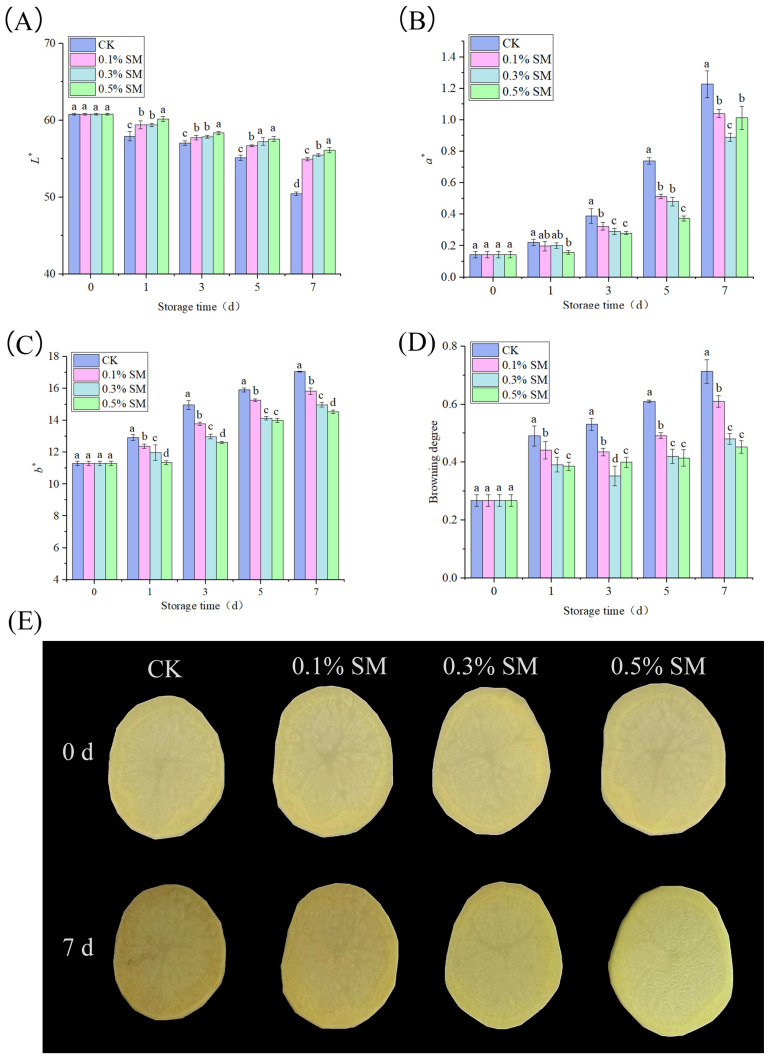
The effects of different concentration of SM treatment on L* value (**A**), a* value (**B**), b* value (**C**), browning degree (**D**) and visual appearance (**E**) in fresh-cut potatoes during storage. Different letters in the columns indicated signifcant differences at *p* < 0.05.

L*, a*, b* indicators are used to evaluate the color change in potatoes. The L* value represents brightness; a higher L* value corresponds to a lighter color and less browning, while a lower L* value indicates greater browning. The a* and b* values indicate red green and yellow blue degrees, respectively, and the higher the a* and b* values, the higher the degree of browning. The L* values showed a decreasing trend throughout the storage period (Figure 1A), while the a* and b* values showed an increasing trend (Figure 1B,C), and the other SM groups showed a slower rate of decrease in the L* values compared to the CK group. After 3 days of storage, the L* values of the CK and SM groups decreased by 6.20%, 5.07%, 4.87%, and 4.01%, respectively, with significant differences between the CK and SM groups (*p* < 0.05). On the 7th day of storage, the L* values of the CK group and 0.1%, 0.3%, 0.5% SM decreased by 17.02%, 9.57%, 7.68%, and 5.92%, and the L* values of the CK group were significantly lower than those of all SM-treated groups (*p* < 0.05). By the end of storage, the CK group exhibited significantly higher a* and b* values compared to the SM-treated groups (*p* < 0.05). This observation of a* and b* values was similar to that of fresh-cut potato slices treated with exogenous chlorogenic acid [19]. This finding suggests that SM markedly influences the color stability of fresh-cut potatoes.

Browning is the primary quality issue that limits the shelf life of fresh-cut potatoes. The cellular integrity of fresh-cut potatoes is disrupted after cutting, and exposure to air accelerates the browning of fresh-cut potato slices [20]. As shown in Figure 1D, the browning degree of fresh-cut potatoes decreased with increasing SM concentration at each time throughout the storage period, with significant differences in browning between the CK and all SM-treated groups (*p* < 0.05). The 0.3% SM and 0.5% SM groups exhibited the lowest browning levels throughout storage. The changes in browning were consistent with the color difference parameters described above. This can be attributed to the fact that SM is a commonly used anti-browning agent, which reduces the accumulation of quinones—key intermediate products in the enzymatic browning pathway—thereby inhibiting browning. Similarly, Oscar Martínez-Alvarez et al. [21] demonstrated that the SM solution effectively delayed the blackening in shrimp. Therefore, SM effectively inhibits browning in fresh-cut potatoes, thereby maintaining a more appealing appearance.

As shown in Figure 1E, the CK and 0.1% SM showed severe browning and developed a slight rancid odor by the end of storage. In contrast, the 0.3% SM and 0.5% SM groups exhibited only slight yellowing and did not show typical browning. Changes in overall appearance and quality were consistent with color differences in fresh-cut potatoes. Similar studies have demonstrated that a 1.5% SM solution can inhibit blackening in giant red shrimp [22]. This effect is mainly attributed to the ability of SM to suppress polyphenol oxidase activity or reduce quinone compounds.

The experimental results showed that the 0.3% SM group and 0.5% SM group could better maintain the appearance quality of fresh-cut potatoes, inhibit the decrease in L* value and inhibit the increase in a* value, b* value and browning degree. SM serves as a common browning inhibitor for fresh-cut produce. Nonetheless, the generation of sulfur dioxide residues from its excessive application remains a safety concern [23]. Therefore, considering cost-effectiveness and food safety, 0.3% SM was selected as the ideal concentration.

### 3.2. Effects of SM on Weight Loss, pH, Soluble Solids, MDA, Total Plate Count Analysis and Sensory Scores of Fresh-Cut Potatoes

**Figure 2:**
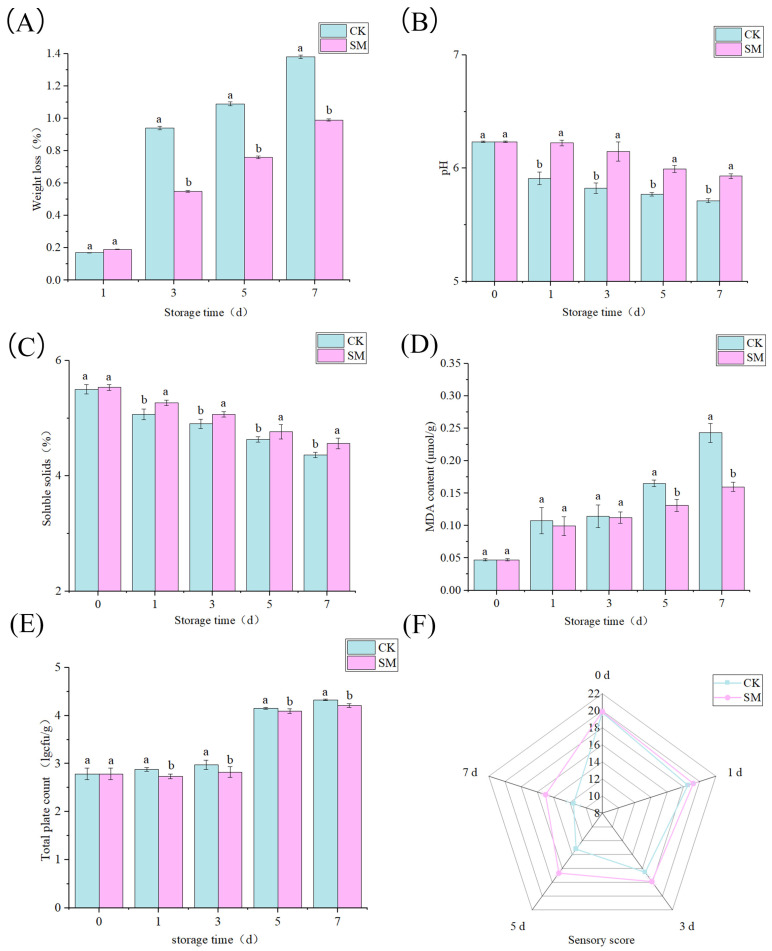
Effects of SM on weight loss (**A**), pH (**B**), soluble solids (**C**), MDA (**D**), total plate count of (**E**) and sensory evaluation (**F**) in fresh-cut potatoes during storage. Different letters in the columns indicated signifcant differences at *p* < 0.05.

The moisture content of fruits and vegetables decreases during storage, so the weight loss becomes a key indicator for assessing the moisture content of food products [24]. Fresh-cut potatoes, owing to their high moisture content and tender tissues, are particularly susceptible to weight loss during chilled storage, mainly due to respiration and transpiration. As shown in Figure 2A, the weight loss of fresh-cut potatoes increased with storage time. This was due to cutting-induced mechanical damage, which partially ruptures the cellular structure, increases the respiration rate, and accelerates water evaporation and weight loss [25]. On the 1st day of storage, no significant difference existed between the SM and CK groups (*p* > 0.05); however, from day 3 to day 7, the SM group showed a significantly lower weight loss rate than the CK group (*p* < 0.05). Especially on the 7th day of storage, the weight loss rates of the SM and CK groups were 0.99% and 1.38%, and the weight loss rate of the CK group was 1.4 times that of SM group. Qasid Alii et al. also showed that SM solution inhibited the increase in the rate of weight loss of pomegranate after harvesting [26]. These findings indicate that SM inhibits the respiration rate of potatoes and reduces respiration-induced water loss, thus effectively suppressing the rate of weight loss.

As shown in Figure 2B, the pH of the SM group was consistently higher than that of the CK group, with significant differences between the two throughout storage. At the end of storage, the pH values of the SM and CK groups were 5.93 and 5.71, respectively, corresponding to decreases of 4.8% and 8.3% from initial values. The decrease in the SM group was significantly lower than that in the CK group (*p* < 0.05). The pH of fresh-cut potatoes gradual decreasing during storage was attributed to respiration, which raised CO_2_ concentration, and the subsequent dissolution of CO_2_ into the cellular liquid medium, thereby increasing acidity [27]. This is due to the fact that SO_2_ in SM can inhibit the respiration rate of fresh-cut potatoes, leading to a decrease in CO_2_ concentration, this is consistent with the previous result that SM inhibited the increase in weight loss of the samples, and suggests that SM can better maintain the flavor of fresh-cut potatoes.

Soluble solids content not only reflects the content of sugar and acid in fruit and vegetable tissues but also reflects the water-holding capacity of cells. Higher soluble solids content enhances cellular osmotic pressure and prevents water from penetrating outside the cell wall. Therefore, the level of soluble solids content directly affects the nutritional quality of fresh-cut potatoes. On day 3 of storage, soluble solids decreased by 11% and 9% in the CK and SM groups (Figure 2C), respectively. On the final day of storage, the soluble solids content of the SM and CK groups were 4.56% and 4.36%, respectively, and the SM group maintained significantly higher soluble solids levels compared to the CK group. It was shown that SM resulted in reduced depletion of soluble solids in fresh-cut potatoes throughout the storage period. This might be related to the fact that SM inhibits the respiration of potatoes, thereby reducing the consumption of sugars.

MDA, the final product of membrane lipid peroxidation, is used as an indicator of cellular damage because lipid peroxidation compromises cell membrane integrity [28]. As shown in Figure 2D, the MDA content of the SM and CK groups increased with storage time, and the increase in MDA was smaller in the SM group and larger in the CK group, which was consistent with the results of browning of fresh-cut potatoes. SM treatment significantly reduced MDA accumulation and delayed membrane damage in fresh-cut potatoes, with MDA levels of 0.16 μmol/g (SM) vs. 0.24 μmol/g (CK) on day 7. These findings confirm that SM effectively delayed membrane damage and reduced MDA accumulation in fresh-cut potatoes, consistent with observations in quercetin-treated potatoes [29].

According to Figure 2E, the total plate count in CK and SM group increased with increasing time, and the total plate count in CK group was significantly higher than that in SM group. On the 3rd day of the chilled storage, the total plate count in SM group and CK group were 2.82 and 3.00 log CFU/g, respectively. At the final point of storage, the total plate count in the SM group was significantly lower than that in the CK group, indicating that the SM solution has antibacterial effect on fresh-cut potatoes. This effect can be attributed to the reducing effect of SM, which can disrupt the normal metabolic oxidation process of microorganisms and inhibit growth and reproduction [30].

Sensory scores, which primarily include color, flavor, texture, and degree of spoilage, can visually reflect the commercial value of fresh-cut potatoes. On day 5 of storage, the average sensory scores of the SM and CK groups were 16.7 and 13.2 (Figure 2F), respectively, and the scores of the CK group were significantly lower than those of the SM group (*p* < 0.05). By day 7, the CK group scored less than 15 points, indicating a loss of commercial value, whereas the SM group retained a sensory score of 15, maintaining commercial value in terms of color, flavor, and texture. These results confirm that SM treatment was effective for potato quality preservation.

### 3.3. Effects of SM on the Texture of Fresh-Cut Potatoes

Firmness is recognized as a key quality indicator of fresh-cut potatoes, which is governed by adhesion and cohesion [31]. The results (Table 1) indicate that the firmness of fresh-cut potatoes decreased gradually over the storage period, and the SM group had significantly higher firmness than the CK group (*p* < 0.05). By the end of storage, the CK group exhibited a firmness of 3329.08 g, whereas the SM group showed 3487.44 g. The reason for this difference might be related to the water loss of the fresh sliced potatoes. The elasticity of CK and SM groups also decreased with storage time, and at the final storage time point, the elasticity of SM and CK groups decreased by 9% and 32.51%, respectively, and the SM group exhibited higher elasticity than the CK group (*p* < 0.05). The chewiness of fresh-cut potatoes also declined over time, with final reductions of 12.10% and 12.83% in the CK and SM groups, respectively, suggesting no statistically significant difference between the two groups. This decline may be associated with moisture loss during storage, which softens tissue structure and reduces firmness and elasticity. SM treatment mitigates water loss, thereby retarding the decrease in firmness and elasticity.

### 3.4. Effects of SM on PPO, POD, and PAL Activities in Fresh-Cut Potatoes

**Figure 3:**
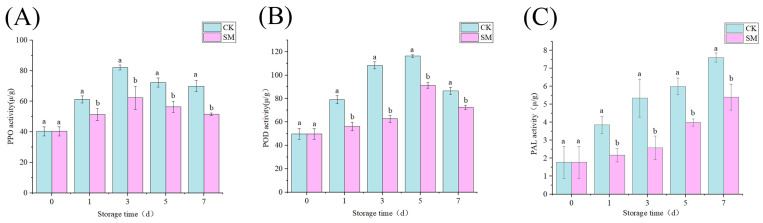
Effects of SM on PPO activity (**A**), POD activity (**B**), and PAL activity (**C**) of fresh-cut potatoes during storage. Different letters in the columns indicated signifcant differences at *p* < 0.05.

Surface browning in fresh-cut produce is directly correlated with the activities of PPO and POD. PPO can catalyze the conversion of phenolic substances into quinones under aerobic conditions, and these quinones can further polymerize to produce black substances, thereby causing browning. POD is a member of the oxidoreductase family, catalyzing the oxidation of phenolic compounds in a hydrogen peroxide-dependent manner [32]. As shown in Figure 3A, PPO activity fluctuated throughout the storage period, initially increasing and then decreasing. On day 3, maximum PPO activity was observed in both groups, with the CK group demonstrating 1.3-fold greater activity relative to the SM group (*p* < 0.05).

POD activity also fluctuated with increasing time (Figure 3B) and reached its maximum on day 5 of preservation. The CK group exhibited significantly higher POD activity than the SM group throughout the entire storage period (*p* < 0.05). On day 7, a 1.2-fold higher POD activity was observed in the CK group compared with the SM group. This increase in enzyme activity can be explained by the mechanical damage sustained by fresh-cut potatoes. Such damage disrupts the balance of reactive oxygen species metabolism, leading to increased activities of PPO and POD. Additionally, when the cell membrane was damaged to a certain extent, the probability of contact between phenolic substrates and enzymes increased, which triggered enzymatic reactions and consequently resulted in browning [33]. The aqueous solution of SM was acidic and contained strong antioxidant components, which reduced the conversion of phenols to quinones and thereby inhibited melanin synthesis, ultimately suppressing PPO and POD activities in fresh-cut potatoes throughout the storage period.

PAL, a wound-inducible enzyme, catalyzes phenylalanine conversion to cinnamic acid, a critical precursor for phenolic compound synthesis. PAL activity is widely used as a marker of phenylalanine metabolic activity [34]. PAL activity progressively increased during storage (Figure 3C). On day 3, the CK group exhibited 2.1-fold higher PAL activity than the SM group. Over the entire storage period, PAL activity in the SM group remained significantly lower than that in the CK group (*p* < 0.05), suggesting that SM treatment inhibited the PAL activity.

### 3.5. Bacterial Community Analysis

Table 2 shows changes in bacterial abundance and alpha diversity during storage under CK and SM treatments. Overall, goods coverage was 1.00 for all samples, indicating that sequencing depth was sufficient to represent the bacterial communities. Across storage time, CK showed fluctuating richness and relatively lower diversity, especially in Shannon and Simpson indices. In contrast, SM generally maintained higher alpha diversity, particularly in community evenness. These results suggest that SM treatment may promote or preserve a more diverse and balanced bacterial community during storage, which might be associated with its inhibitory effect on dominant bacterial groups.

**Figure 4:**
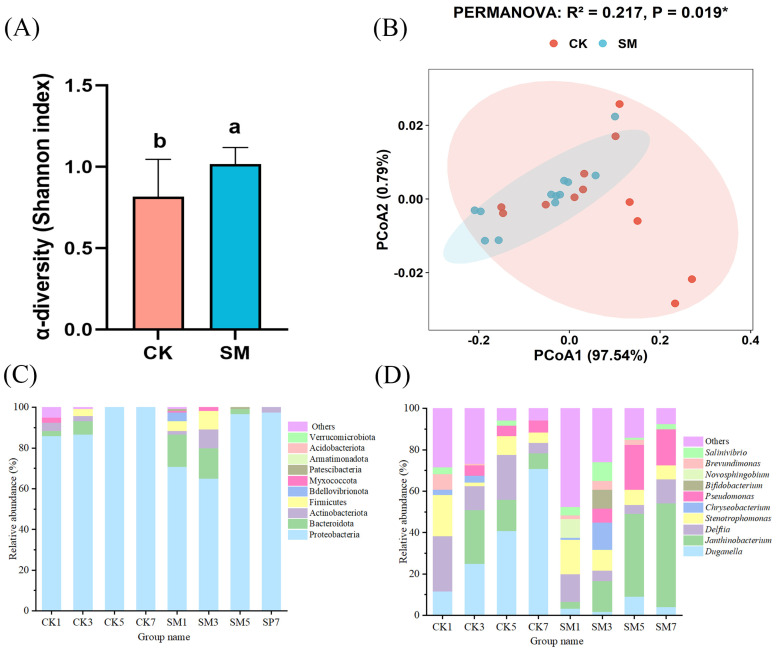
The analysis of alpha-diversity (**A**), beta-diversity between CK group (red area) and SM group (blue area) (**B**), bacterial community at the phylum level (**C**) and genus level (**D**) during storage at 4 °C in fresh-cut potatoes in the SM and CK groups. Different letters in the columns (**A**) and the * (**B**) indicated signifcant differences at *p* < 0.05.

The Shannon index was significantly higher in the SM group than in the CK group (Figure 4A), indicating that SM treatment increased the diversity of the microbial community. Consistent with the α-diversity results, principal coordinate analysis (PCoA) revealed a clear shift in microbial community composition between the two groups (Figure 4B). The first two principal coordinates explained 97.54% and 0.79% of the total variation, respectively. Although some overlap was observed, samples from the SM group tended to cluster separately from those of the CK group. Furthermore, PERMANOVA analysis confirmed that the overall microbial community structure differed significantly between the two groups (R^2^ = 0.217, *p* = 0.019), suggesting that SM treatment not only enhanced microbial diversity but also markedly reshaped the microbial community composition. To further elucidate the shifts in bacterial community and abundance, the microbial composition was analyzed at both the phylum and genus levels. As shown in Figure 4C, the dominant phyla were Proteobacteria, Bacteroidota, Actinobacteriota, and Firmicutes. This finding is consistent with the results of Jiang et al. [35], who investigated the effects of vacuum and modified-atmosphere packaging on the microbial community dynamics of fresh-cut potatoes. Throughout the storage period, the dominant bacteria in the SM and CK groups were Proteobacteria and Bacteroidota. In the CK group, Proteobacteria showed a gradual increase. In the SM group, Proteobacteria exhibited a trend of first decreasing and then increasing, falling from 70.83% on day 1 to 65% on day 3, and then rising to 97.5% on day 7. Proteobacteria are common microorganisms in plants and are generally found mainly on the surface of plant tissues, which may explain their high abundance in fresh-cut potatoes. In both the CK and SM groups, Bacteroidota decreased over time, and by the end of the storage period, its abundance had dropped to almost 0% in both groups.

At the genus level (Figure 4D), on the first day, the dominant bacteria in the SM and CK groups were *Duganella*, *Janthinobacterium*, *Delftia*, and *Stenotrophomonas*. *Janthinobacterium* abundance in the SM group increased from 3.3% to 50% over 7 days, while in the CK group it peaked at 25.8% on day 3 and dropped to 7.5% by day 7. Conversely, *Duganella* abundance in the SM group peaked at 9.2% on day 5 and then declined, whereas in the CK group it steadily rose from 11.7% to 70.8%. *Janthinobacterium* and *Duganella* are widely known for their antifungal activities, they are Gram-negative, motile, aerobic bacteria commonly isolated from soil and aquatic environments, both genera belong to the family Oxalobacteraceae [36,37]. Notably, certain *Duganella* species possess the ability to produce amylase [38], which may contribute to tissue softening and structural degradation in fresh-cut potatoes during storage. Interestingly, while SM treatment suppressed *Duganella*, the relative abundance of *Janthinobacterium* showed a slight increase. Several possibilities may explain this observation. *Janthinobacterium* species are known to produce violacein, a purple-pigmented secondary metabolite with broad-spectrum antimicrobial and antifungal properties [39]. The enrichment of *Janthinobacterium* in SM-treated samples might represent a competitive release effect, wherein the suppression of competing taxa by SM creates a niche that favors the proliferation of *Janthinobacterium*. At the end of storage, the relative content of *Delftia* in the SM and CK groups was 11.7% and 5%, respectively. *Delftia* is a motile, aerobic, non-budding Gram-negative bacterium primarily found in environments such as river water and soil [40].

In the SM group, the relative abundance of *Stenotrophomonas* decreased over time and remained relatively stable in the later stages of storage, whereas in the CK group, it fluctuated throughout the storage period. This fluctuation may be associated with the growth of *Duganella*, which dominated the microbial community in the CK group. *Stenotrophomonas* is a Gram-negative, conditionally pathogenic bacterium that is commonly found in the natural environment, primarily in animals, plants, soil, and water sources [41]. The genus *Stenotrophomonas* merits particular attention in the context of food safety, as certain species—most notably *Stenotrophomonas maltophili* (*S. maltophilia*)—are recognized as opportunistic pathogens in immunocompromised individuals, associated with nosocomial infections and multidrug resistance [42]. In this study, *Stenotrophomonas* was detected in both the CK and SM groups, with its relative abundance varying over time. However, several points should be considered when interpreting this finding. First, the presence of *Stenotrophomonas* in fresh-cut potatoes does not necessarily indicate a health risk, as the pathogenicity of this genus is primarily clinically relevant and typically requires a susceptible host and high inoculum levels [43]. Second, the species-level identity of the *Stenotrophomonas* sequences detected in our samples remains unknown, as the 16S rRNA gene amplicon sequencing approach used in this study does not provide sufficient resolution to distinguish between *S. maltophilia* and other non-pathogenic species within the genus. Third, the relative abundance of *Stenotrophomonas* in both treatment groups was relatively low and declined over time, particularly in the SM group. Nonetheless, we acknowledge that the potential presence of opportunistic pathogens in fresh-cut potatoes warrants caution, and future studies employing species-level identification methods are needed to more accurately assess the microbiological safety of SM-treated fresh-cut potatoes.

### 3.6. Residual SO_2_ in Fresh-Cut Potatoes

**Figure 5:**
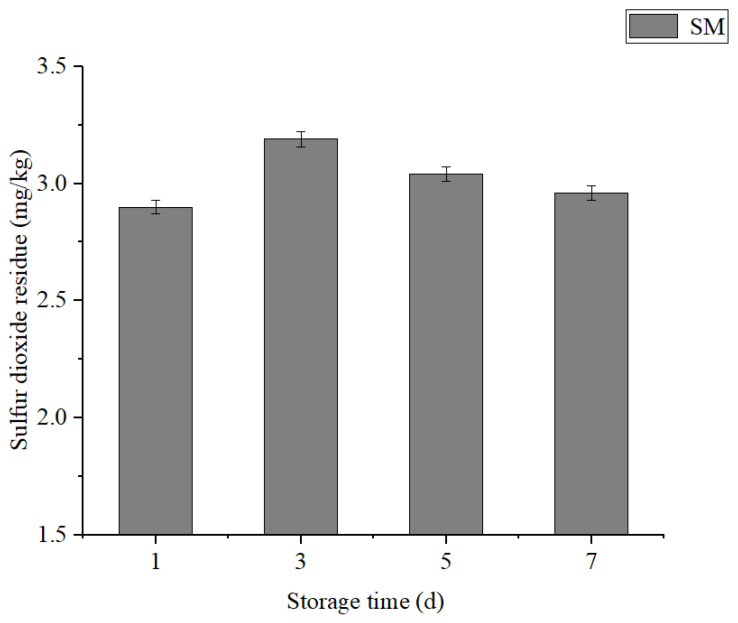
SO_2_ residues in fresh-cut potatoes during the storage period.

In food systems, sulfites serve as effective preservatives; however, they may provoke allergy-like responses in susceptible individuals, with symptoms varying from mild skin rashes and nausea to severe respiratory distress [44]. The main substance of sodium metabisulfite residue in food is sulfur dioxide (SO_2_). As shown in Figure 5, the SO_2_ residue in fresh-cut potatoes fluctuated between 2.96 mg/kg and 3.19 mg/kg, and there was no SO_2_ residue limit standard for fresh-cut potatoes in the Chinese standard. According to the Chinese standard GB 2760-2024 [45], the maximum residue limit of SO_2_ in surface-treated fresh fruit and vegetable juice was 50 mg/kg. The U.S. Food and Drug Administration (FDA) has established a maximum residue limit of 10 mg/kg for sulfite in food, expressed as sulfur dioxide [46]. In the European Union, Regulation (EC) No 1169/2011 requires label declaration of sulfites when levels exceed 10 mg/kg in the final product, reflecting a consumer protection approach focused on allergen labeling. Given that the SO_2_ residue in SM-treated fresh-cut potatoes ranged from 2.96 to 3.19 mg/kg, this level was substantially lower than both the Chinese standard (50 mg/kg) and the U.S. FDA limit (10 mg/kg).

## 4. Conclusions

The results of this experiment demonstrated that SM treatment could delay the browning of fresh-cut potatoes and maintain a superior appearance and nutritional quality compared to the CK group. SM treatment maintained the textural properties of fresh-cut potatoes, such as firmness and elasticity, inhibited the reduction in PPO, POD, and PAL activities and the growth of *Duganella*, reduced MDA accumulation, and maintained cell membrane integrity. These findings suggest that SM treatment could serve as a practical and reliable method for preserving the quality of fresh-cut potatoes. However, several limitations should be acknowledged. SM efficacy may vary with cultivar, microbial load, and storage conditions. Although SO_2_ residues (2.96–3.19 mg/kg) were below regulatory limits (China: 50 mg/kg; U.S. FDA: 10 mg/kg), sulfite-sensitive consumers may still be at risk, and label declaration may be required in some markets.

## Figures and Tables

**Table 1:** Effects of SM on the texture of fresh-cut potatoes over storage.

Indicators	Group	Storage Time (d)				
		0	1	3	5	7
Firmness (g)	CK	4002.39 ± 101.89 ^a^	3722.38 ± 30.88 ^b^	3579.13 ± 127.55 ^b^	3481.11 ± 112.83 ^b^	3329.08 ± 75.10 ^b^
	SM	4002.39 ± 101.89 ^a^	3874.17 ± 53.38 ^a^	3743.51 ± 31.14 ^da^	3617.60 ± 145.30 ^a^	3487.44 ± 78.44 ^a^
Elasticity (mm)	CK	0.80 ± 0.04 ^a^	0.80 ± 0.04 ^a^	0.77 ± 0.03 ^a^	0.78 ± 0.06 ^a^	0.54 ± 0.02 ^b^
	SM	0.81 ± 0.03 ^a^	0.83 ± 0.04 ^a^	0.80 ± 0.06 ^a^	0.80 ± 0.07 ^a^	0.72 ± 0.03 ^a^
Chewines (mJ)	CK	1439.98 ± 56.73 ^a^	1385.30 ± 43.73 ^b^	1393.50 ± 65.63 ^a^	1375.72 ± 88.82 ^a^	1265.68 ± 44.10 ^a^
	SM	1528.12 ± 69.76 ^a^	1532.55 ± 129.17 ^a^	1447.07 ± 150.42 ^a^	1395.79 ± 47.54 ^a^	1332.05 ± 75.10 ^a^

Notes: All values are expressed as the mean (*n* ≥ 3) ± standard deviation. Different letters in the same column mean significant difference at *p* < 0.05.

**Table 2:** Analysis of bacterial abundance and alpha diversity analysis of samples during storage.

Storage Time (d)	Group Name	Chao1	Goods_Coverage	OTUs	Shannon	Simpson
1	CK	30.40 ± 6.77 ^a^	1 ^a^	30 ± 6 ^a^	0.80 ± 0.31 ^a^	0.33 ± 0.19 ^a^
	SM	50.17 ± 33.12 ^a^	1 ^a^	52 ± 28 ^a^	1.00 ± 0.01 ^a^	0.43 ± 0.01 ^a^
3	CK	46.28 ± 34.88 ^a^	1 ^a^	43 ± 30 ^a^	0.83 ± 0.11 ^b^	0.32 ± 0.06 ^b^
	SM	25.258 ± 4.99 ^a^	1 ^a^	25 ± 5 ^a^	1.09 ± 0.11 ^a^	0.48 ± 0.05 ^a^
5	CK	30.50 ± 4.34 ^a^	1 ^a^	30 ± 5 ^a^	0.89 ± 0.18 ^a^	0.37 ± 0.11 ^a^
	SM	35.04 ± 4.57 ^a^	1 ^a^	35 ± 5 ^a^	1.03 ± 0.10 ^a^	0.45 ± 0.05 ^a^
7	CK	34.73 ± 12.79 ^a^	1 ^a^	35 ± 12 ^a^	0.75 ± 0.37 ^a^	0.29 ± 0.22 ^a^
	SM	34.00 ± 10.83 ^a^	1 ^a^	33 ± 10 ^a^	0.94 ± 0.13 ^a^	0.39 ± 0.10 ^a^

Data are presented as mean ± SD (*n* = 3). Within the same storage day, different superscript letters indicate significant differences among treatment groups at *p* < 0.05.

## Data Availability

The original contributions presented in this study are included in the article. Further inquiries can be directed to the corresponding authors.
